# Intravenous Tranexamic Acid Reduces Post-Operative Bleeding and Blood Transfusion in Patients Undergoing Aortic Surgery: A PRISMA-Compliant Systematic Review and Meta-Analysis

**DOI:** 10.31083/j.rcm2404120

**Published:** 2023-04-19

**Authors:** Bo Zhang, Li-xian He, Yun-tai Yao, the Evidence in Cardiovascular Anesthesia (EICA) Group

**Affiliations:** ^1^Department of Anesthesiology, Tianjin Union Medical Center, 300121 Tianjin, China; ^2^Department of Anesthesiology, Fuwai Yunnan Cardiovascular Hospital, 650000 Kunming, Yunnan, China; ^3^Department of Anesthesiology, Fuwai Hospital, National Center for Cardiovascular Diseases, Peking Union Medical College and Chinese Academy of Medical Sciences, 100037 Beijing, China

**Keywords:** tranexamic acid, aortic surgery, post-operative bleeding

## Abstract

**Background:**

Tranexamic acid (TXA), an antifibrinolytic agent, has been demonstrated to reduce blood loss and transfusion requirements in both cardiac and non-cardiac surgery. However, the evidence regarding the efficacy of intravenous TXA in aortic surgery has been seldomly analyzed. Therefore, the current study was performed to address this question.

**Methods:**

Searches of PubMed, EMBASE, OVID, Cochrane Library and CNKI were conducted comprehensively for randomized controlled trials (RCTs) comparing intravenous TXA versus no-TXA. Independently and in duplicate, we reviewed titles, abstracts and full-text articles, extracted data and evaluated bias risks. A random effect or fixed effect model was utilized to pool data.

**Results:**

The database search yielded 4 RCTs involving 273 patients. Meta-analysis revealed that, there was a significant reduction in bleeding volume within the first 4 hours post-operatively [(weighted mean difference (WMD) = –74.33; 95% confidence interval (CI): –133.55 to –15.11; *p* = 0.01)], and the first 24 hours post-operatively [(WMD = –228.91; 95% CI: –352.60 to –105.23; *p* = 0.0003)], post-operative red blood cell (RBC) transfusion volume [(WMD = –420.00; 95% CI: –523.86 to –316.14; *p *< 0.00001)], fresh frozen plasma (FFP) transfusion volume [(WMD = –360.35; 95% CI: –394.80 to –325.89; *p *< 0.00001)] and platelet concentrate (PC) transfusion volume [(WMD = –1.27; 95% CI: –1.47 to –1.07; *p *< 0.0001)] following intravenous TXA administration. In addition, intravenous TXA administration significantly decreased the incidence of postoperative complications (53/451 (8.2%) vs. 75/421 (13.9%); odds ratio (OR) = 0.47; 95% CI: 0.30 to 0.75; *p* = 0.001), according to this present meta-analysis.

**Conclusions:**

The current study preliminarily demonstrated that, TXA significantly reduced postoperative bleeding, blood transfusion requirements and postoperative complications among patients undergoing aortic surgery. More well-designed studies are warrant to confirm the efficacy and safety of intravenous TXA in patients undergoing aortic surgery.

## 1. Introduction

Aortic aneurysm surgery was first performed by Cooley in 1952 [1]. This type of surgery is commonly accompanied by excessive bleeding after surgery, which often requires extensive blood transfusion. Numerous factors contribute to this problem. Activation of the fibrinolytic system secondary to rapidly formed thrombi, extracorporeal circulation, deep hypothermia and circulatory arrest (DHCA) [2, 3] have been certified as an important mechanism for surgery-related bleeding. And all these factors complicated post-operative patient outcomes.

The lysine analogue tranexamic acid (TXA) was discovered by Shosuke and Utako in 1962 [4]. At present, TXA is a frequently-used antifibrinolytic agent in cardiac and non-cardiac surgeries. Although tranexamic acid had a significant effect on reduction of bleeding in previous researches, its role in aortic surgery was rarely studied [5, 6, 7, 8, 9, 10, 11]. The efficacy of TXA in aortic surgical patients remains unknown. Therefore, the current study was performed to address this question.

## 2. Materials and Methods

### 2.1 Search Strategy

As outlined in **Supplementary Table 1**, we conducted a systematic review according to Preferred Reporting Items for Systematic Reviews and Meta-Analysis Guidelines (PRIMSA) [12]. CRD42020186673 was the registration number for the protocol of the current meta-analysis. PubMed, EMBASE, OVID, and Cochrane Library were computerized searched to identify all relevant studies till May 1st, 2020, with different combination of search terms used as follows: (“Tranexamic acid” OR “AMCHA” OR “trans-4-(Aminomethyl) cyclohexanecarboxylic Acid” OR “AMCA” OR “t-AMCHA” OR “Cyklokapron” OR “Anvitoff” OR “KABI 2161” OR “Ugurol” OR “Transamin” OR “Spotof” OR “Exacyl” OR “Amchafibrin”) AND (“Extracorporeal circulation” OR “Cardiopulmonary bypass” OR “Cardiovascular surgical procedures” OR “Aortic aneurysm” OR “Aortic dissection” OR “deep hypothermic circulatory arrest”) AND (“controlled clinical trial” OR “randomized controlled trial” OR “placebo” OR “randomly” OR “trial” OR “randomized”) (Appendix Table 2). Chinese BioMedical Literature & Retrieval System (till May 1st, 2020) were likewise searched. Language restriction was not used.

### 2.2 Inclusion Criteria

Our study included all randomized controlled trials (RCTs) comparing TXA with no treatment or placebo in patients undergoing open aortic surgery in terms of efficiency and safety. Tranexamic acid, no treatment and placebo groups were eligible only from studies with other comparator drugs. The primary outcomes were the intra- and post-operative blood loss, allogeneic transfusion and incidence of reoperation due to post-operative bleeding. The secondary outcomes were the incidences of myocardial infarction (A new Q wave on the electrocardiogram, a creatine kinase MB/creatine kinase ratio greater than 10%, and a troponin I value of more than 0.1 ng/dL on the troponin I test) [6], stroke, acute lung injury (hypoxemia occurred within 72 h after surgery, an oxygenation index (arterial oxygen partial pressure/inhaled oxygen concentration) <150 mmHg) [13], pulmonary infection, acute renal insufficiency (two times the baseline creatinine level or dialysis needed) [6], gastrointestinal bleeding, hepatic insufficiency (glutamate-pyruvate transaminase (GPT) >200 U/L, total bilirubin (TBIL) >50 mm/L, and lactate dehydrogenase (LDH) >50 U/L occurring within one week after surgery, with or without clinical manifestations of liver insufficiency) [14].

### 2.3 Exclusion Criteria

Exclusion criteria included: (1) articles published in case reports, abstracts, or reviews; (2) observation or retrospective studies (3) articles without information on outcomes of interest; (4) cell or animal studies; (5) duplicate publications. Titles and abstracts of all the identified eligible studies were independently reviewed to exclude evidently ineligible ones by two authors (BZ and LXH). We further determined whether eligibility for these residual studies was ultimately included by reading the full article.

### 2.4 Study Quality Assessment

The risk of bias was assessed independently by two authors (BZ and LXH), using the tool referred to in the Cochrane Handbook for Systematic Reviews of Interventions [15]. Further, two authors (LXH and YTY) independently evaluated the methodological quality of each trial using the modified 7-point Jadad score [16]. The trials with 1–3 points were evaluated as low quality, and the trials with 4–7 points were evaluated as high quality.

### 2.5 Data Abstraction

The following data were independently extracted by two authors (BZ and LXH) from the included studies: (1) author, countries and publication years of studies included; (2) total number of participants, age, sex, body weights (BW)/body mass indexes (BMI) of patients in TXA and placebo/blank groups; (3) surgical type; (4) data related to the outcomes of interest in two groups. Data processing was completed with all disagreements resolved through discussion among all authors.

### 2.6 Statistical Analysis

The datum was analyzed using RevMan 5.3 (Cochrane Collaboration, Oxford, UK). For dichotomous data, we used a pooled odds ratio (OR) and 95% confidence interval (CI), while for continuous data, we used weighted mean difference (WMD) and 95% CI. Depending on whether significant heterogeneity (*$I^{2}$* >50%) existed for each outcome, randomized-effects or fixed-effects models were utilized. We examined how statistical models affected estimates of management effects in sensitivity analyses. All analyses adopted the fixed-effects model were twice assessed via randomized-effects model and vice versa. Furthermore, sensitivity analyses were conducted to exam the effect of individual studies on the overall results. An investigation of the publication bias was conducted using funnel plots of the outcomes. In all cases, the *p*-value was two-sided, and *p *< 0.05 was considered statistically significant.

## 3. Results

### 3.1 Search Results

**Fig. 1.**
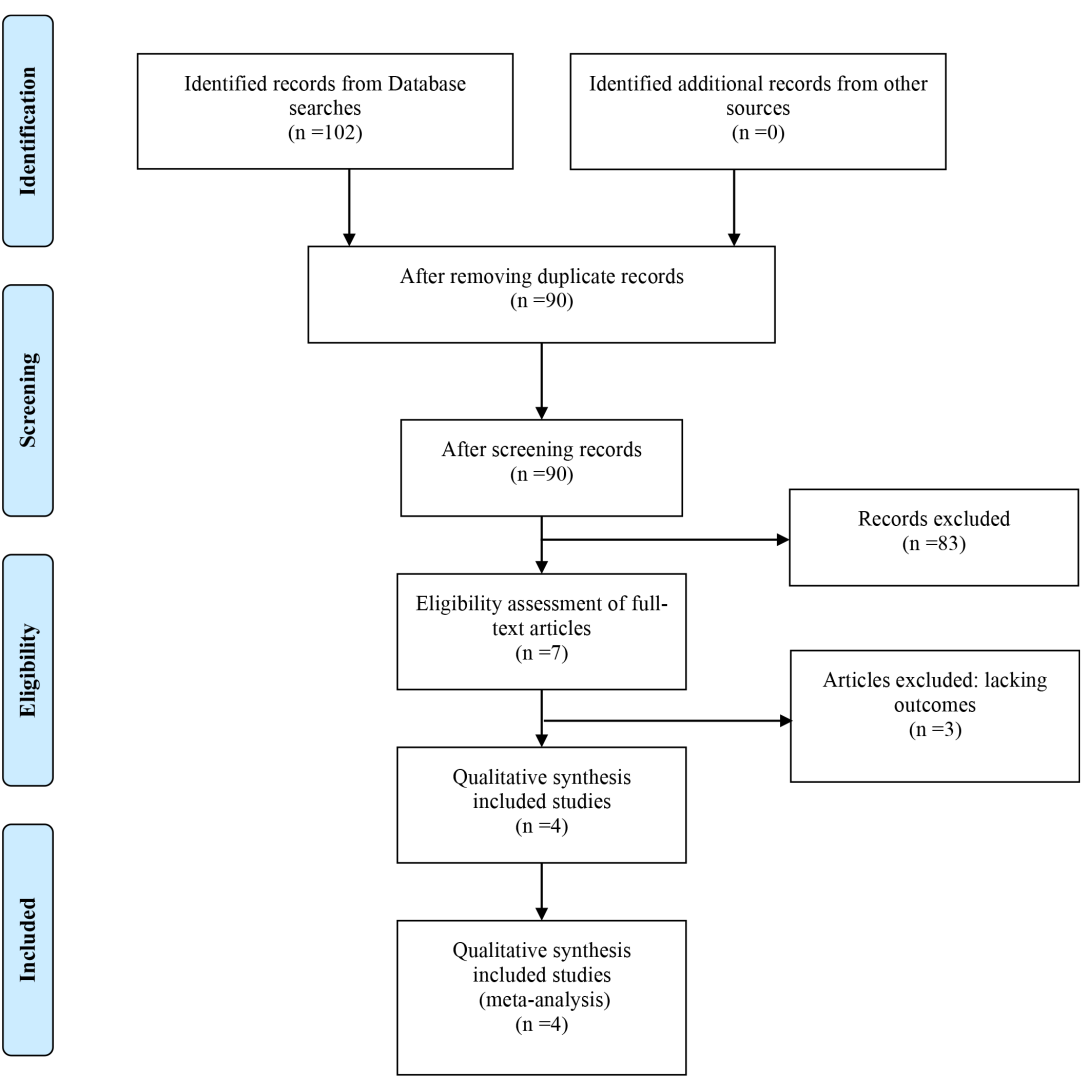
**PRISMA flow diagram**.

Fig. 1 illustrates how database searches identified seven articles for full evaluation. There were four studies eligible for inclusion in the meta-analysis [6, 8, 10, 11]. Table 1 (Ref. [6, 8, 10, 11]) presents a description of these articles. Of the 4 references, two [6, 8] were written in English (2 from Italy), and the other two [10, 11] were in Chinese.

**Table 1.** **Characteristics of included trials**.

Trials	Country	Surgery	Group TXA					Group Control					Outcomes
			Dosage	n	Age (yr)	Sex (M/F)	BW/BMI	Control	n	Age (yr)	Sex (M/F)	BW/BMI	
Casati 2002 [6]	Italy	TAA surgery	1 g (20 min)* iv *before SI, then 400 mg/h till EOP, 0.5 g in CPB prime	29	59 ± 13	23/6	74 ± 14/	NS	29	63 ± 11	19/10	76 ± 17/	①②③④⑤⑦⑨⑪⑫⑬⑭⑮
Monaco 2020 [8]	Italy	AAA surgery	0.5 g *iv* 20 min before SI, then 250 mg/h till EOP	50	69 ± 9	43/7	/25.3 ± 3.3	NS	50	69 ± 7	50/0	/26.2 ± 2.6	①②③④⑤⑦⑭⑮
Xu 2015 [10]	China	TAA surgery	10 mg/kg *iv* before SI, then 10 mg/kg/h till EOP	30	45 ± 10	21/9	/25.1 ± 2.7	NS	26	47 ± 9	18/8	/25.5 ± 3.6	②③⑥⑧⑩⑫⑬⑮
Zhang 2016 [11]	China	TAA surgery	10 mg/kg (30 min) *iv* before SI, then 10 mg/kg/h till EOP	30	42 ± 4	26/5	44 ± 4/	NS	29	41 ± 4	27/4	43 ± 4/	②③⑥⑧⑩⑫⑬⑭⑮

AAA, abdominal aortic aneurysm; BW, body weight; BMI, body mass index; CPB, cardiopulmonary bypass; F, female; *iv*, intravenously; M, male; EOP, end of operation; SI, skin incision; TAA, thoracic aortic aneurysm; TXA, tranexamic acid; yr, year; Outcomes ①, first postoperative 4-hour blood bleeding volume; ②, first postoperative 24-hour blood bleeding volume; ③, reoperation for bleeding; ④, intra-operative RBC transfusion rate; ⑤, intra-operative FFP transfusion rate; ⑥, post-operative RBC transfusion volume; ⑦, post-operative RBC transfusion rate; ⑧, post-operative FFP transfusion volume; ⑨, post-operative FFP transfusion rate; ⑩, post-operative PC transfusion volume; ⑪, post-operative PC transfusion rate; ⑫, mechanical ventilation; ⑬, intensive care unit stay; ⑭, hospital stay; ⑮, post-operative complications.

### 3.2 Included Trials Characteristics

As showed in Table 1, among the four trials, three included patients undergoing thoracic aortic aneurysm surgery [6, 10, 11], one included patients with surgery of abdominal aortic aneurysm [8]. There were 273 patients enrolled in the 4 eligible trials, and 139 were allocated to the TXA group and 134 to the Control (placebo) group.

### 3.3 Study Quality and Risk Bias

**Supplementary Figs. 1,2** show the bias risk analysis. Randomization was utilized in all 4 trials [6, 8, 10, 11], with double blindness in two [6, 8], single blindness in two [10, 11]. Three trials [6, 8, 10] described patient withdrawal or dropouts, and one [11] left out the reason for the withdrawal or dropout. There were four trials with unclear reporting bias [6, 8, 10, 11], and one trial with unclear attrition bias [11]. Four included RCTs received modified Jadad scores of 3 to 7, with 1 RCT [11] rated “low quality” (1–3 points) and three RCTs [6, 8, 10] rated “high quality” (4–7 points).

### 3.4 Effects on Post-Operative Bleeding

**Fig. 2.**
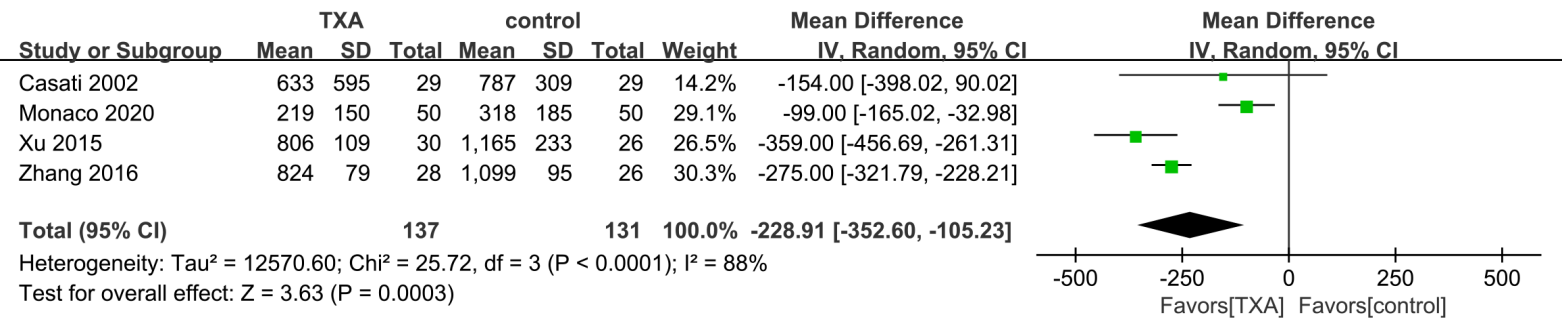
**First postoperative 24-hour bleeding volume**. TXA, tranexamic acid.

Based on Table 1, 4 trials [6, 8, 10, 11] (4 comparisons, 273 patients) reported re-operations for bleeding, 2 trials [6, 8] (2 comparisons, 158 patients) reported blood loss in first 4-hour post-operatively, 4 trials [6, 8, 10, 11] (4 comparisons, 273 patients) reported blood loss in first 24-hour post-operatively. Re-operation for bleeding (**Supplementary Fig. 3**), first 4-hour (**Supplementary Fig. 4**) and first 24-hour (Fig. 2) post-operatively blood loss was compared between Group TXA and Group Control. TXA administration significantly reduced the rate of re-operations for bleeding [(2/137 (1.5%) vs. 11/131 (8.4%); OR = 0.25; 95% CI: 0.07 to 0.82; *p* = 0.02), with no heterogeneity (*$I^{2}$* = 0%, *p* = 0.42)], the first 4 hours [(WMD = –74.33; 95% CI: –133.55 to –15.11; *p* = 0.01) with heterogeneity (*$I^{2}$* = 81%, *p* = 0.02)] and the first 24 hours post-operative bleeding volume [(WMD = –228.91; 95% CI: –352.60 to –105.23; *p* = 0.0003) with heterogeneity (*$I^{2}$* = 88%, *p *< 0.00001)].

### 3.5 Effects on Post-Operative Blood Transfusion

The data in Table 1 were reported in two trials [10, 11] (two comparisons, 110 patients). Red blood cell (RBC) transfusion volume is shown in Fig . 3, fresh frozen plasma (FFP) transfusion volume is shown in **Supplementary Fig. 5**, and platelet concentrate (PC) transfusion volume is shown in **Supplementary Fig. 6**. Meta-analysis revealed that there was a significant reduction in RBC [(WMD = –420.00; 95% CI: –523.86 to –316.14; *p *< 0.00001) with no heterogeneity (*$I^{2}$* = 0%, *p *< 1.00)], FFP [(WMD = –360.35; 95% CI: –394.80 to –325.89; *p *< 0.00001) with no heterogeneity (*$I^{2}$* = 0%, *p* = 0.89)], and PC [(WMD = –1.27; 95% CI: –1.47 to –1.07; *p *< 0.00001) with no heterogeneity (*$I^{2}$* = 0%, *p* = 0.65)] transfusion volumes after TXA administration following surgical procedures.

**Fig. 3.**
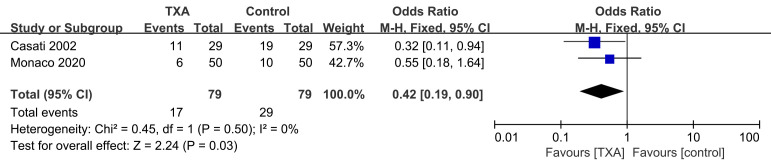
**Postoperative RBC transfusion volume**. TXA, tranexamic acid; RBC, red blood cell.

Table 1 reports data from two trials [6, 8] (2 comparisons, 110 patients) concerning post-operative RBC transfusion rate (**Supplementary Fig. 7**). Meta-analysis demonstrated that TXA decreased the need for post-operative RBC transfusions [(17/79 (21.5%) vs. 29/79 (36.7%); OR = 0.42; 95% CI: 0.19 to 0.90; *p* = 0.03) with no heterogeneity (*$I^{2}$* = 0%, *p* = 0.50)]. Based on the data in Table 1, one trial [6] (58 patients) reported post-operative FFP transfusion rate (9/29 (31.0%) vs. 15/29 (51.7%); OR = 0.42, 95% CI: 0.14 to 1.23; *p* = 0.11) and post-operative PC transfusion rate (1/29 (3.4%) vs. 1/29 (3.4%); OR = 1.00, 95% CI: 0.06 to 16.79; *p* = 1.00), which was similar between TXA group and control group.

### 3.6 Effects on Post-Operative Complications

**Fig. 4.**
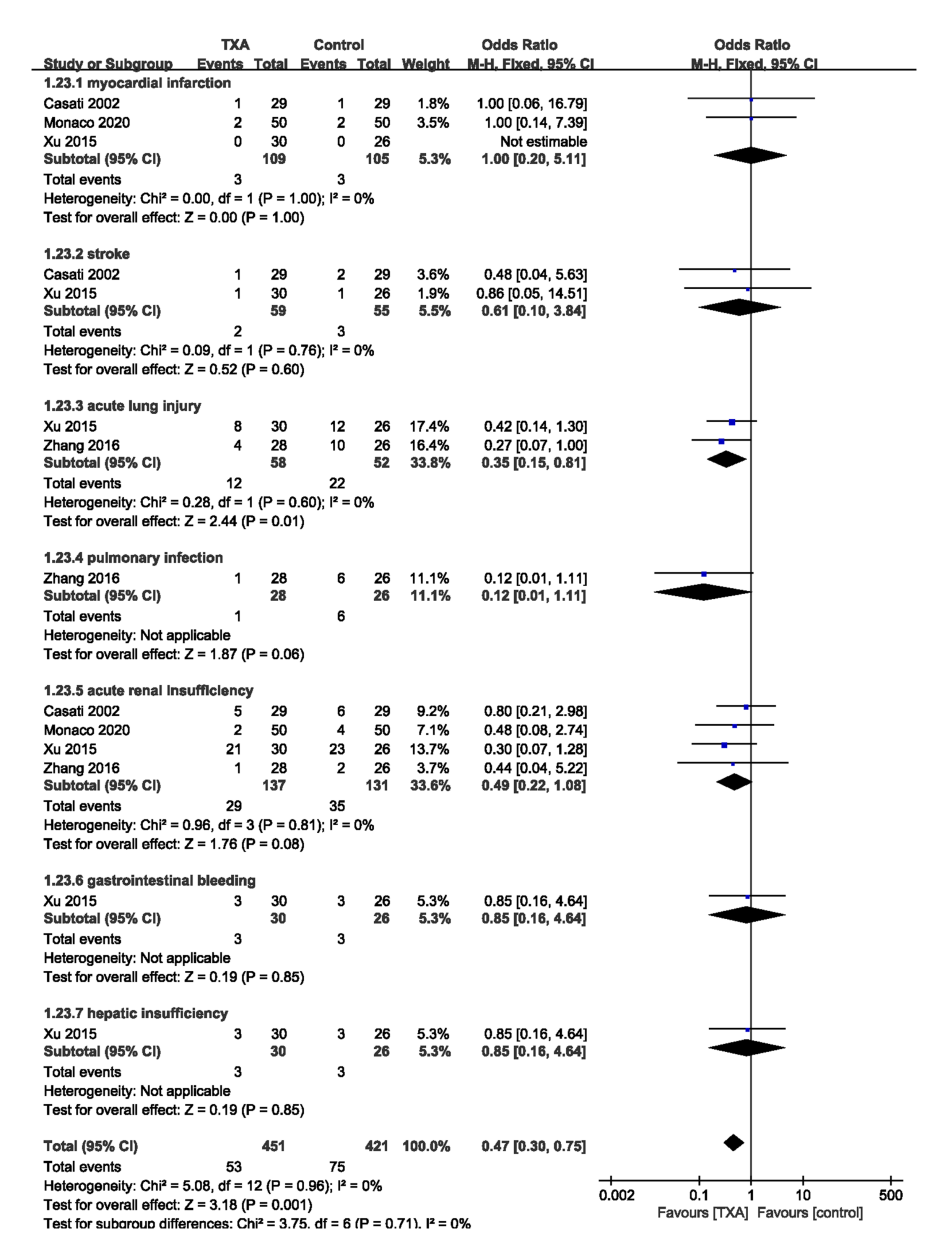
**Incidence of postoperative complications**. TXA, tranexamic acid.

Post-operative complications were identified in four trials [6, 8, 10, 11] (273 patients) (Fig. 4). Meta-analysis demonstrated that that group TXA significantly reduced the incidence of post-operative complications (a composite outcome variably defined by individual study authors) [(53/451 (8.2%) vs. 75/421 (13.9%); OR = 0.47; 95% CI: 0.30 to 0.75; *p* = 0.001) with no heterogeneity (*$I^{2}$* = 0%, *p* = 0.96)].

One trial [10] reported that there was no difference between the TXA group and the control group in terms of gastrointestinal bleeding (3/30 (10.0%) vs. 3/26 (11.5%), *p* = 0.85) and hepatic insufficiency (3/30 (10.0%) vs. 3/26 (11.5%), *p* = 0.85).

One trial [11] reported the incidence of pulmonary infection (1/28 (3.6%) vs. 6/26 (23.1%), *p* = 0.06) was similar among TXA group and control group participants.

Two trials [6, 10] (114 patients) reported the incidence of stroke [(2/59 (3.4%) vs 3/55 (5.5%); OR = 0.61; 95% CI: 0.10 to 3.84; *p* = 0.60) with no heterogeneity (*$I^{2}$* = 0%, *p* = 0.76)] was similar among TXA group and control group participants.

Three trials [6, 8, 10] (214 patients) reported there was no difference between the TXA group and the control group in terms of myocardial infarction [(2.8% vs. 2.9%; OR = 1.00; 95% CI: 0.20 to 5.11; *p* = 1.00) with no heterogeneity (*$I^{2}$* = 0%, *p* = 1.00)].

Four trials [6, 8, 10, 11] (273 patients) reported the occurrences of acute renal insufficiency [(21.2% vs. 26.7%; OR = 0.49; 95% CI: 0.22 to 1.08; *p* = 0.08) with no heterogeneity (*$I^{2}$* = 0%, *p* = 0.81)] were similar among TXA group and control group participants.

Two trials [10, 11] reported the incidence of acute lung injury in 110 patients. Meta-analysis demonstrated that, TXA administration was associated with a lower incidence of acute lung injury [(12/58 (20.7%) vs. 22/52 (42.3%); OR = 0.35; 95% CI: 0.15 to 0.81; *p* = 0.01) with no heterogeneity (*$I^{2}$* = 0%, *p* = 0.60)].

### 3.7 Sensitivity Analyses and Publication Bias

According to sensitivity analysis, statistical model choice did not affect treatment effects. In addition, sensitivity tests were conducted to determine how individual studies influence overall effects by removal of each study. For high heterogeneity outcomes, we also performed sensitivity tests to examine the influences of the overall treatment effects on it by exclusion of some studies, whereas we did not find any contradictory results. A funnel plot analysis of primary and secondary outcomes did not reveal any significant publication bias.

## 4. Discussion

As far as we know, this present meta-analysis is the initial study to analysis the effects of intravenous TXA on perioperative blood loss, perioperative blood transfusion and postoperative complications in aortic surgery patients. The results indicated that intravenous TXA administration significantly reduced the volume of post-operative bleeding within the first 4 hours and the first 24 hours and transfusion requirement in aortic surgery. Also, intravenous TXA administration significantly decreased the incidence of postoperative complications according to this present meta-analysis.

Aortic surgery involves high levels of bleeding and perioperative blood transfusions as a result, which frequently complicates patient outcomes. The preoperative factors include the diseased aortic fragile tissues due to the formation of a false lumen and increased fibrinolysis caused by the tissue factors and then activate coagulation factor vii to start the extrinsic coagulation pathway [17]. Several intraoperative factors are also involved, as well as a large surgical surface, the application of extracorporeal circulation, and DHCA [18, 19]. All these factors play parts in the complex changes of hemostatic and the consequent abnormal perioperative blood loss frequently occurred in patients undergoing aortic surgery. TXA is an antifibrinolytic agent which is widely used in the management of reduction of intra-and postoperative bleeding and transfusion requirements. It has been certified that TXA can reduce blood loss and transfusion requirements effectively in cardiac [20] and non-cardiac surgical patients [21, 22]. Similar results were obtained by Ahn *et al*. [5] in a study which retrospectively studied data from 55 adult patients who underwent acute aortic dissection surgery between April 2008 and April 2010. Moreover, Makhija *et al*. [7] also reported TXA had equal effect in reducing the perioperative bleeding and transfusion requirements in patients undergoing surgery for thoracic aortic compared with epsilon-amino-caproic acid. Similar findings [6, 8, 10, 11] supported the conception that the intravenous injection of TXA played a significant role in the reduction of postoperative bleeding and transfusion requirement after aortic surgery.

Our meta-analysis also showed that no significant difference was detected in the rate of FFP and platelets postoperatively transfusion among the groups. However, in the rate of reoperation for bleeding and postoperative transfusion of RBC, TXA group showed significantly less rate compared to placebo. Casati *et al*. [6], Xu* et al*. [10] and Zhang *et al*. [11] demonstrated similar results in their respective studies comparing the groups. Decreasing operative bleeding would lessen the risks and costs related to blood transfusion. In addition, a higher rate of surgical reexploration occurred in patients with excessive bleeding [23]. Contrarily, some trials [5, 6, 7, 8] reported that TXA administration had no effect on reducing the rate of reoperation for bleeding or the rate of postoperative transfusion of RBC in aortic surgery. It is assumed that a much larger sample size would be necessary to detect potential differences between groups of related outcomes. Hopefully further well-designed trials will address this issue in a more comprehensive way.

The current study demonstrated that, intravenous TXA was associated with lower incidences of postoperative complications [6, 8, 10, 11]. Furthermore, no differences between the groups were observed in terms of thromboembolic complications (myocardial infarction or stroke). As we known, it is associated with poorer postoperative outcomes when excessive bleeding leads to blood transfusions. In fact, it has been proven in two large observational studies that perioperative transfusions are associated with increased rates of morbidity and mortality during the 30 days following major vascular surgery [24, 25]. In addition, an allogenic blood supply is limited and should only be used when absolutely necessary. Thus, Strategies or drugs that can reduce bleeding and transfusion requirements may improve clinical outcomes. However, the use of tranexamic acid was still controversial, because sporadic reports of thrombotic events and stroke was reported. Recently, an analysis of over 5000 cardiac surgery patients showed that tranexamic acid administration was not related with a higher risk of thrombotic complications [26], which was consistent with the results of the present study. However, Zhou *et al*. [27] reached the opposite conclusion in a retrospective study that the use of intraoperative TXA during cardiac surgery was associated with postoperative strokes.

Several limitations are present in this study. It cannot be denied that, by pooling a large number of small, low-quality studies, the meta-analysis is able to expand the power of the analysis, but its limitations are also evident. These include multiple TXA dosages, limited trial scale and quality and heterogeneity issues of included studies. We aimed to provide some evidence about this clinical controversy with our meta-analysis. The only way to prove this is through more well-designed, large-scale randomized trials.

## 5. Conclusions

The present study provided some preliminary evidence that intravenous administration of TXA in aortic surgical patients was effective not only in reducing blood loss and transfusion requirement, but also so in lowering the incidences of postoperative complications.

## Supplementary Material

Supplementary material associated with this article can be found, in the online version, at https://doi.org/10.31083/j.rcm2404120.

## Appendix

See Table 2.

**Table 2.** **Database search strategies**.

Search strategies		Results
**PubMed**		15
	**#1**: (“tranexamic acid”[MeSH Terms] OR (“tranexamic”[All Fields] AND “acid”[All Fields]) OR “tranexamic acid”[All Fields]) AND ((“aorta”[MeSH Terms] OR “aorta”[All Fields] OR “aortic”[All Fields]) AND (“surgery”[Subheading] OR “surgery”[All Fields] OR “surgical procedures, operative”[MeSH Terms] OR (“surgical”[All Fields] AND “procedures”[All Fields] AND “operative”[All Fields]) OR “operative surgical procedures”[All Fields] OR “surgery”[All Fields] OR “general surgery”[MeSH Terms] OR (“general”[All Fields] AND “surgery”[All Fields]) OR “general surgery”[All Fields])) AND (Randomized Controlled Trial[ptyp] AND “humans”[MeSH Terms])	
	**#2**: (“tranexamic acid”[MeSH Terms] OR (“tranexamic”[All Fields] AND “acid”[All Fields]) OR “tranexamic acid”[All Fields]) AND (“aortic aneurysm”[MeSH Terms] OR (“aortic”[All Fields] AND “aneurysm”[All Fields]) OR “aortic aneurysm”[All Fields]) AND (Randomized Controlled Trial[ptyp] AND “humans”[MeSH Terms])	
	**#3**: ((“tranexamic acid”[MeSH Terms] OR (“tranexamic”[All Fields] AND “acid”[All Fields])) OR “tranexamic acid”[All Fields]) AND ((((“aneurysm, dissecting”[MeSH Terms] OR (“aneurysm”[All Fields] AND “dissecting”[All Fields])) OR “dissecting aneurysm”[All Fields]) OR (“aortic”[All Fields] AND “dissection”[All Fields])) OR “aortic dissection”[All Fields])	
	**#4**: (“tranexamic acid”[MeSH Terms] OR (“tranexamic”[All Fields] AND “acid”[All Fields]) OR “tranexamic acid”[All Fields]) AND (“aorta”[MeSH Terms] OR “aorta”[All Fields]) AND (Randomized Controlled Trial[ptyp] AND “humans”[MeSH Terms])	
	**#5**: #1 OR #2 OR #3 OR #4	
**OVID**		10
	**#1**: aortic surgery Including Limited Related Terms	
	**#2**: limit #1 to original articles	
	**#3**: aortic dissection Including Limited Related Terms	
	**#4**: limit #3 to original articles	
	**#5**: aortic aneurysm Including Limited Related Terms	
	**#6**: limit #5 to original articles	
	**#7**: #2 OR #4 OR #6	
	**#8**: tranexamic acid Including Limited Related Terms	
	**#9**: limit #8 to original articles	
	**#10**: #7 AND #9	
**Cochrane**		2
	**#1**: (tranexamic acid):ti,ab,kw	
	**#2**: (aortic surgery):ti,ab,kw OR (aortic aneurysm):ti,ab,kw (aortic dissection):ti,ab,kw	
	**#3**: (human):ti,ab,kw	
	**#4**: #1 AND #2 AND #3	
**EMBASE**		68
	(‘aortic surgery’:ti,ab,kw OR ‘aortic aneurysm’:ti,ab,kw OR ‘aortic dissection’:ti,ab,kw OR aorta:ti,ab,kw) AND ‘tranexamic acid’:ti,ab,kw	
**CKNI**		7
	((SU = 主动脉) OR (KW = 主动脉) OR (SU = 主动脉瘤) OR (KW = 主动脉瘤) OR (SU = 主动脉夹层) OR (KW = 主动脉夹层)) AND ((SU = 氨甲环酸) OR (KW = 氨甲环酸))	
