# Supplementary material for: Intravenous Tranexamic Acid Reduces Post-Operative Bleeding and Blood Transfusion in Patients Undergoing Aortic Surgery: A PRISMA-Compliant Systematic Review and Meta-Analysis
Source: Rev Cardiovasc Med. 2023 Apr 19;24(4):120. doi: 10.31083/j.rcm2404120 (PMC11273041; doi:10.31083/j.rcm2404120)
Supplement: Supplementary file 1 [file 2153-8174-24-4-120-s1.zip › Supplementary Figures.docx]

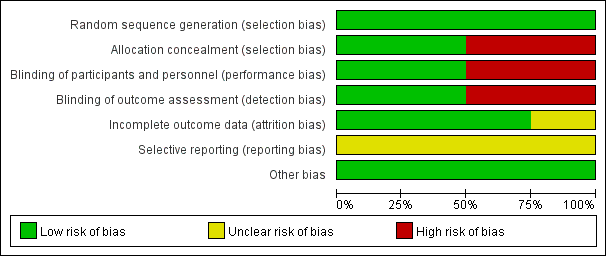


Supplementary Fig. 1. Risk of bias graph.


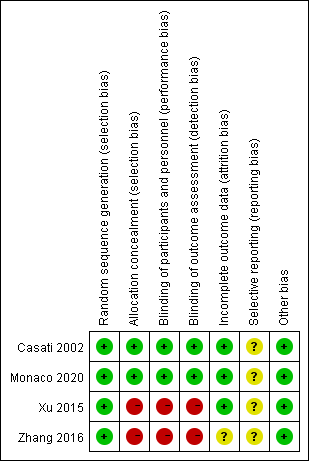


Supplementary Fig. 2. Risk of bias summary.


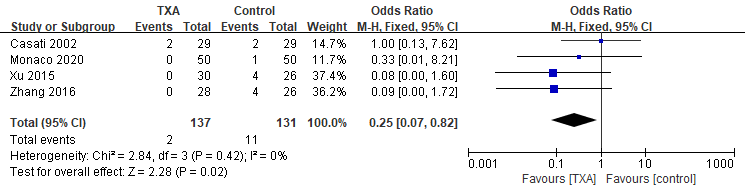


Supplementary Fig. 3. Re-operation for bleeding.


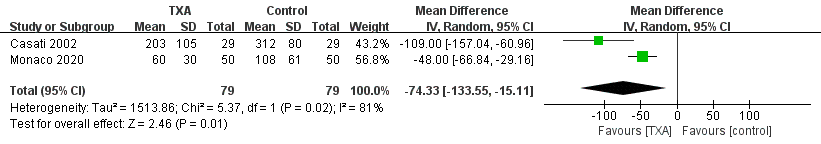


Supplementary Fig. 4. First postoperative 4-hour bleeding volume.


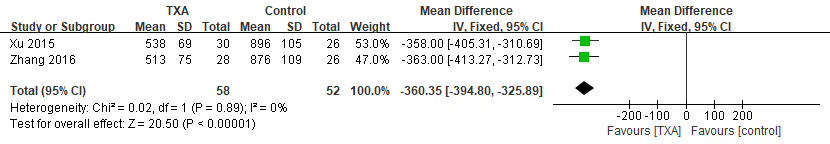


Supplementary Fig. 5. Postoperative FFP transfusion volume.


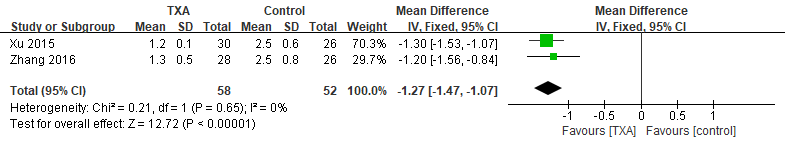


Supplementary Fig. 6. Postoperative PC transfusion volume.


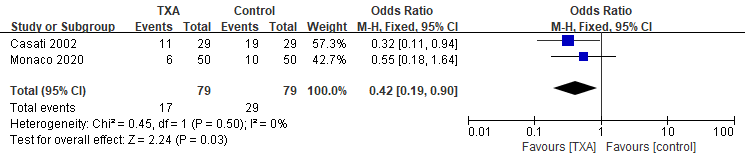


Supplementary Fig. 7. Postoperative RBC transfusion rate.
